# What Sex-Disaggregated Metrics Are Needed to Explain Sex Differences in COVID-19?

**DOI:** 10.3389/fgwh.2020.00002

**Published:** 2020-07-03

**Authors:** Carinna Hockham, Kelly Thompson, Cheryl Carcel, Ana-Catarina Pinho-Gomes, Mark Woodward, Sanne A. E. Peters

**Affiliations:** ^1^The George Institute for Global Health, University of New South Wales, Sydney, NSW, Australia; ^2^The George Institute for Global Health, University of Oxford, Oxford, United Kingdom; ^3^Julius Center for Health Sciences and Primary Care, University Medical Center Utrecht, Utrecht University, Utrecht, Netherlands

**Keywords:** Coronavirus COVID-19, sex-disaggregated data, sex differences, surveillance data, global health

Between early December 2019 and June 28 2020, there have been around 9.9 million confirmed cases of COVID-19 and 498,895 related deaths in 187 countries (1). In nearly all countries where sex-disaggregated data are available, men who are diagnosed with COVID-19 appear more likely than women to experience severe disease and eventually die from it (2), although the relative and absolute difference in reported case fatality rates between women and men varies between countries. This finding has attracted attention in the scientific community, and media more broadly (3). Current hypotheses to explain this observation center around differences, to men's disadvantage, in the prevalence of pre-existing chronic disease comorbidities and lifestyle risk factors, such as personal hygiene, smoking and alcohol consumption, immunological differences, and genetic factors (4).

The volunteer-led Global Health 50/50 initiative is tracking the availability of sex-disaggregated COVID-19 data on the numbers of confirmed cases and deaths. As of June 28 2020, they report data from 133 countries, representing 99% of global confirmed cases and >99% of reported deaths. Of these, 40% (*n* = 53) report sex-disaggregated data on *both* cases and deaths and 37% (*n* = 49) report *either* cases or deaths (Figure 1). At the time of writing, the 60% of countries that do not report sex-disaggregated data on both metrics contain more than half (53%) of the reported global burden of COVID-19 deaths and account for half of the global population. Although Global Health 50/50 does not capture all countries where COVID-19 cases have been identified, including some reporting sex-specific data, these data suggest a significant gap in sex-disaggregated COVID-19 surveillance data.

**Figure 1:**
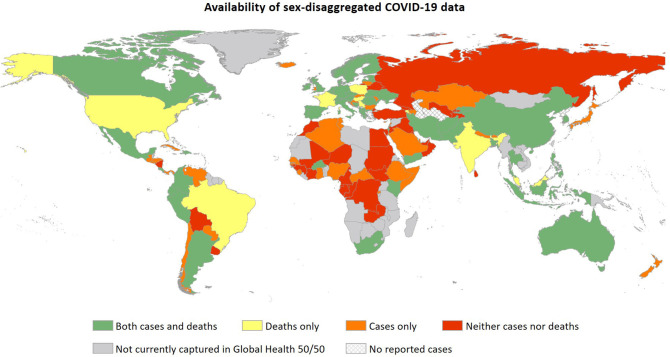
Availability of sex-disaggregated data on confirmed COVID-19 cases and deaths in countries represented in the Global Health 50/50 COVID-19 sex-disaggregated data tracker on June 15 2020 (https://globalhealth5050.org/covid19/).

Overall and sex-disaggregated data on the numbers of confirmed cases and deaths are undoubtedly useful for assessing the magnitude of the pandemic. However, the data available to date make it difficult to accurately quantify sex differences in COVID-19 infection and mortality rates. For instance, testing criteria in many countries prioritize healthcare workers (the majority of whom are women), potentially resulting in more cases being identified in women who are at otherwise relatively low mortality risk. On the other hand, other key workers who have a high risk of exposure to COVID-19 infection (e.g. social workers), and are disproportionately likely to be female (5), are, unfortunately, not prioritized. If sufficiently large, these sex differences in testing may limit the comparability of case fatality rates in women and men. Another nuance to the current data in countries experiencing a high burden of COVID-19 is that reported deaths often only include those occurring in hospital. Without an accurate count of all COVID-19-attributable deaths, disaggregated by sex (and ideally age and ethnicity), care should be taken when quantifying sex differences.

Other sex-disaggregated surveillance data that would facilitate a better understanding of how COVID-19 differentially affects women and men include hospital and intensive care unit (ICU) admissions and lengths of stay, as well as the provision of invasive ventilation and other types of organ support. Such data would enable better determination of the extent to which the risk of severe disease is lower for women, or whether women do experience severe disease but are more likely to survive. This has important prognostic implications and would increase understanding of disease progression in women and men as well as the longer-term needs of COVID-19 survivors, many of whom may experience respiratory, cardiovascular and/or renal complications, either as acute events or due to pre-existing conditions becoming exacerbated. This could result in a substantial deterioration in mental and physical health, as is commonly seen in survivors of sepsis (6).

As we continue to broaden our understanding of COVID-19, we must also be cognizant of the need for well-designed sex and gender COVID-19 research that represents all patients. To do this well, a proactive intersectionality-informed approach to research design and data analysis is crucial (7). Data from Italy and the UK suggest that the overall prevalence of comorbidities in those who die from COVID-19 is similar for women and men (8, 9). However, the *type* of comorbidities varies, with pre-existing heart failure, hypertension, dementia and autoimmune diseases being more common in women, and ischemic heart disease, liver disease and chronic kidney disease more common in men. This likely reflects known, and in part age-related, sex differences in the prevalence of these comorbidities in the general population, but it does raise important questions around whether clinical management of COVID-19 should incorporate a sex lens. Moreover, based on experiences from previous infectious disease epidemics, we must plan to record and examine the pregnancy status of COVID-19-infected women so that pregnancy and perinatal outcomes of COVID-19 can be more fully understood early on. All this must be done against the backdrop of intersecting factors, including gender, age, ethnicity, and socioeconomic status.

The COVID-19 pandemic has illuminated well-known, yet all too often neglected, health disparities based on sex, gender, race, ethnicity, and socioeconomic status (10, 11). For sex, we know from other disease areas that differences in clinical presentation, disease progression and treatment outcomes between women and men have historically been overlooked and that this has cost lives (12). This must not be repeated. In the current pandemic, it is imperative that sex-disaggregated data are collected and effectively analyzed from the outset so that policies that appropriately address the needs of both women and men can be developed.

If any countries with sex-disaggregated data are not currently represented in the Global Health 50/50 database and would like to be, please contact info@globalhealth5050.org.

## Author Contributions

CH, KT, and SP drafted the manuscript. All authors provided critical revision for important intellectual content.

## Conflict of Interest

The authors declare that the research was conducted in the absence of any commercial or financial relationships that could be construed as a potential conflict of interest.
